# Materials of the 2nd Conference with International Participation “Basic Research in Endocrinology: A Modern Strategy for the Development and Technologies of Personalized Medicine”

**DOI:** 10.3390/jpm15040139

**Published:** 2025-04-01

**Authors:** Oksana Rymar, Alla Ovsyannikova, Elena Shakhtshneider

**Affiliations:** Research Institute of Internal and Preventive Medicine—Branch of the Institute of Cytology and Genetics, Siberian Branch of Russian Academy of Sciences, Borisa Bogatkova Str. 175/1, 630089 Novosibirsk, Russia

## 1. Introduction

There has been an increase in patients with diabetes mellitus (DM) all over the world, with the advent of new modern research methods (for example, molecular genetic diagnostics), non-classical types of diabetes are increasingly being identified [1,2]. These types include MODY (Maturity Onset Diabetes of the Young). One of the days of the 2nd Conference with International Participation, “Basic Research in Endocrinology: A Modern Strategy for the Development and Technologies of Personalized Medicine”, was devoted to this topic. On another day, presentations were given on the most relevant topics in endocrinology, including various thyroid diseases, modern diagnosis and therapy of diabetes, neuroendocrine diseases, reproductive health, and more.

This Special Issue features several articles, the data of which were presented in the conference reports. The articles describe the results of studies on type 1 diabetes mellitus (T1DM), type 2 diabetes mellitus (T2DM), MODY, and sarcopenia, as well as reviews on obesity and type 1 interferon antibodies. The abstracts of these articles are presented below.

## 2. Review of Published Articles

In the article by Danil E. Kladov et al. (contribution 1), the development of nocturnal hypoglycemia depending on nocturnal glucose profiles in T1DM was described. A total of 395 patients underwent indirect glucose monitoring, during which ten clusters without hypoglycemia and six clusters with episodes of nocturnal hypoglycemia were identified. Predictors of decreased glucose were determined in clusters without hypoglycemia. The results demonstrated the diversity of nocturnal glucose profiles in patients with T1DM, which underscores the need for a differentiated approach to prescribing insulin therapy.

Evgeniya V. Garbuzova et al. (contribution 2) reported in their article the prevalence of T2DM in a cohort study of people aged 25–44 years and also identified risk factors for diabetes in this age group. The prevalence of DM was 0.82%. Patients with T2DM had a larger waist circumference; higher body mass index, systolic blood pressure, and triglyceride levels; and lower HDL levels than patients without T2DM. They were also less likely to have higher education. The risk of developing T2DM increases 6.5-fold with a BMI of ≥30 kg/m^2^ and 5.2-fold with a triglyceride level of ≥1.7 mmol/L, regardless of other risk factors. In the absence of higher education, the risk of developing T2DM increases 5.6-fold. Thus, the levels of these indicators should be taken into account in young people, and when they increase, the individuals should be screened for the presence of T2DM.

In the article by Dinara Ivanoshchuk et al. (contribution 3), the genetic aspects of MODY diabetes are described in detail. One patient and his relatives were diagnosed with a pathogenic variant in the *ABCC8* gene and in the *HNF1a* gene, which makes it possible to plan the therapy for and prevention of specific complications for these patients.

Yulia G. Samoilova et al. (contribution 4) demonstrated in their article the importance of pro-inflammatory markers in the prognostic diagnosis of sarcopenia. The study included two groups: the main group consisted of 146 participants, and the control group consisted of 75 participants. A twofold increase in nitrates was detected in the main group, and a negative relationship between nitrate levels with weak grip strength and appendicular muscle mass was determined in the main group, adjusted for multiple variables. The results of this study can be used to develop a screening method for the diagnosis of sarcopenia at the outpatient stage.

In the article by Sofia Malyutina et al. (contribution 5), glucose-lowering therapy was analyzed in a random sample of the population (n = 3898, comprising both men and women aged 55–84) in Novosibirsk in 2015–2018 (HAPIEE project). Among patients with T2DM, 59% of individuals received hypoglycemic therapy, and 32% did not. Glycemic control (fasting plasma glucose < 7.0 mmol/L) was achieved in every fifth participant with T2DM (35% of those who received therapy). In terms of the frequency of use of hypoglycemic therapy, biguanides ranked first (75%), sulfonylurea derivatives ranked second (35%), insulins ranked third (12%), and inhibitors of DPP4 ranked fourth (5%). In a sample of the population aged 55–84 years, examined in 2015–2018, glycemic control was achieved in every fifth participant with T2DM and in every third participant who received therapy. The data obtained show a low percentage of achieving the target glycemia levels in elderly patients.

Vladimir B. Berikov et al. (contribution 6) developed machine learning-based models for the short-term prediction of nocturnal hypoglycemia in patients with T1DM. The models were created based on continuous glucose monitoring data, and eight parameters from this study were included. Combinations of continuous monitoring parameters and clinical data (23 parameters) were also evaluated. Basal insulin dose, duration of diabetes, proteinuria, and HbA1c were the most important clinical predictors of nocturnal hypoglycemia. Machine learning is a modern method for predicting various conditions, including hypoglycemia.

In an article by Ahmad Bairqdar et al. (contribution 7), variants of genes related to adipocyte function, as well as variants of genes associated with metabolic aberrations and concomitant disorders in visceral obesity, are considered. It is known that genetic predisposition and the influence of environmental factors contribute to the development of obesity. The article presents an extensive analysis of changes in the structure and functional activity of the genes encoding adipocytokines.

Xavier Eugenio León Aguilera et al. (contribution 8) analyzed the data on the influence of microbiota on the development of obesity. Dysbiosis was recognized as one of the many factors associated with obesity characterized by the predominance of Firmicutes, a decrease in Bifidobacterium in the intestine, and a subsequent decrease in the synthesis of short-chain fatty acids, which led to a decrease in the action of incretins and increased intestinal permeability. Bacteria, bacterial endotoxins, and toxic bacterial products enter the bloodstream, leading to systemic inflammation associated with obesity. The authors examined these relationships in detail, as well as the effects of pro- and prebiotics on them.

Nurana Nuralieva et al. (contribution 9) compared three different methods—multiplex micromatrix, cellular, and enzyme immunoassays—to detect antibodies to omega-interferon and alpha2-interferon. Only a cell-based immunoassay can determine the neutralizing activity of autoantibodies; a microarray-based immunoassay can serve as a highly specific and sensitive screening test to identify patients with positive results for antibodies to interferon 1.

In an article by Gerson Fabián Gualdrón-Bobadilla et al. (contribution 10), the changes occurring in the stomatognathic system of patients with obesity following bariatric surgery were studied. Studies published between 2010 and October 2021 in the main databases were reviewed. An analysis of changes and structures in patients with obesity and candidates for bariatric surgery showed that changes in the stomatognathic system during the preoperative period are understandable due to the availability of a wide range of information. However, information remains limited regarding the postoperative period. Therefore, further research is needed, focusing on the characteristics of the system after surgery.

## 3. Conclusions

This collection of articles on topical issues in endocrinology covers modern methods of diagnosis and therapy of common endocrine diseases. The articles describe the results of research on common endocrine pathologies (type 1 and type 2 diabetes mellitus) and rare diseases (MODY and sarcopenia), which is of interest not only to endocrinologists but also to doctors of other specialties. 
